# IL-32 induces epithelial-mesenchymal transition by triggering endoplasmic reticulum stress in A549 cells

**DOI:** 10.1186/s12890-020-01319-z

**Published:** 2020-10-23

**Authors:** Ling Gong, Gang Liu, Honglan Zhu, Caihong Li, Pengmei Li, Changlu Liu, Hongbo Tang, Kaifeng Wu, Jie Wu, Daishun Liu, Xiaoping Tang

**Affiliations:** 1 The First Clinical Medical College, Jinan University, 601 W. Huangpu Avenue, Guangzhou, 510630 China; 2 Department of Respiratory Medicine, The Third Affiliated Hospital of Zunyi Medical University (The First People’s Hospital of Zunyi), Zunyi, 563000 Guizhou China; 3 Institute of Respiratory Diseases, The Second Affiliated Hospital of Army Medical University (Third Military Medical University), Chongqing, 400037 China; 4 Zunyi Medical University, Zunyi, 563000 Guizhou China; 5 Scientific Research Center, The Third Affiliated Hospital of Zunyi Medical University (The First People’s Hospital of Zunyi), Zunyi, 563000 Guizhou China; 6 Department of Respiratory, The Third Affiliated Hospital of Zunyi Medical University (The First People’s Hospital of Zunyi), No.98 Fenghuang Road, Zunyi, 563002 Guizhou China

**Keywords:** Idiopathic pulmonary fibrosis, Epithelial-mesenchymal transition, Endoplasmic reticulum stress, IL-32

## Abstract

**Background:**

Epithelial-mesenchymal transition (EMT) is a key process in the onset and development of idiopathic pulmonary fibrosis (IPF) with unclear mechanisms. Our previous studies found that bleomycin and tunicamycin could induce ER stress and consequently trigger EMT accompanying with IL-32 overexpression. This study was aimed to investigate the effects of IL-32 on EMT and ER stress to elucidate the pathogenesis of IPF.

**Methods:**

Human lung adenocarcinoma A549 cells were treated with recombinant human (rh)IL-32, IL-32 siRNA and EMT inducer tunicamycin, or 4-phenylbutyric acid (4-PBA), respectively. Then the cell morphology was observed and the expression of ER-related markers and EMT-related markers were detected by RT-qPCR or western blotting.

**Results:**

Stimulation of A549 cells with rhIL-32 led to a morphological change from a pebble-like shape to an elongated shape in a portion of the cells, accompanied by down regulated expression of the epithelial cell marker E-cadherin and up regulated expression of the mesenchymal cell markers N-cadherin, Vimentin, and Zeb-1. However, these rhIL-32 induced changes were inhibited by the ER stress inhibitor 4-PBA. Suppression of IL-32 expression with siRNA inhibited TM-induced EMT. Further stimulation of the A549 cells with rhIL-32 demonstrated an increase in the expression of GRP78, although this increase was also inhibited by 4-PBA.

**Conclusions:**

These results suggest that IL-32 induces EMT in A549 cells by triggering ER stress, and IL-32 may be a novel marker for IPF.

**Supplementary information:**

**Supplementary information** accompanies this paper at 10.1186/s12890-020-01319-z.

## Background

Idiopathic pulmonary fibrosis (IPF), a chronic progressive lung disease characterized by continuous scarring of the lungs, can lead to a progressive decline of lung function and respiratory failure, with consequently high mortality rates [1]. The incidence of IPF has shown an increasing trend in recent years. However, since the precise cause and pathogenic mechanism are still unknown, there is a lack of effective treatment measures for IPF. Consequently, the median survival of IPF patients after diagnosis is only 2–3 years, and the mortality rate is higher than that of many cancers [2]. Therefore, in-depth investigations of the molecular mechanisms of IPF are of great significance for guiding the development of novel strategies for the prevention and treatment of the disease.

The onset of IPF involves disruption of the apoptosis/proliferation balance of fibroblasts, along with the excessive synthesis and accumulation of extracellular matrix components such as collagen [3]. Myofibroblasts are the primary source of collagen production, which are mainly regulated by the epithelial-mesenchymal transition (EMT) [4], a phenomenon in which epithelial cells acquire the phenotypic and biological characteristics of mesenchymal cells in response to certain stimuli [5]. Detailed studies have demonstrated that epithelial cells that have undergone EMT possess a contractile function and the ability to synthesize collagen [4], which are the key conditions for the onset and development of fibrosis. Thus, EMT appears to play an important role in IPF pathogenesis.

Recent research has also demonstrated a link between the EMT and endoplasmic reticulum (ER) stress. Certain factors such as smoking, chronic aspiration, and viral infection may induce ER stress in type II alveolar epithelial cells (AEC II), leading to the dissociation of the ER chaperone glucose-regulated protein 78 (GRP78), thus blocking its downstream effectors to ultimately result in cell apoptosis and the induction of EMT [6, 7]. In addition, 4-phenylbutyric acid (4-PBA) was shown to inhibit the activation of ER stress and induction of EMT in AEC II cells [8–10]. We also previously reported that bleomycin and tunicamycin (TM) could induce ER stress and consequently trigger EMT through activation of histone deacetylase with accompanying interleukin (IL)-32 overexpression [11].

IL-32 is a recently discovered cytokine that plays a pivotal role in innate and acquired immunity through the regulation of T cells [12]. IL-32 can participate in the onset and development of many types of tumors by influencing the EMT processes of tumor cells. For example, IL-32α can inhibit JAK2 / STAT3 signaling pathway to inhibit IL-6-induced EMT and tumor cell invasion and metastasis in pancreatic cancer cells [13]. IL-32beta could increase the production of VEGF and resulted in increasement of cell migration and invasion through STAT3 activation [14]. IL-32θ was reported to inhibit JAK2 / STAT3 signaling pathway and thereby reduce IL-6 induced EMT production [15], it also lead to the inhibition of epithelial-mesenchymal transition (EMT) and stemness by reserving STAT3-ZEB1 pathway [16]. Accordingly, we hypothesized that IL-32 might play a role in the pathogenic mechanism of IPF by influencing EMT. To test this possibility, we evaluated the role of IL-32 in A549 lung adenocarcinoma cells and the mechanisms of action in relation to the influence on EMT and ER stress. The results of this study could provide a scientific basis for IL-32 as a novel target in future research and clinical treatments of IPF.

## Methods

### Cell culture and treatment

Human lung adenocarcinoma A549 cells are an alveolar epithelial cell line with biological characteristics of AEC II, and were therefore used as an in vitro model of IPF. The A549 cells (ATCC^R^CRM-CCL-185™) were purchased from American Type Culture Collection, and cultured in RPMI-1640 culture medium containing 10% fetal bovine serum, 100 U/mL penicillin, and 100 μg/mL streptomycin in a constant-temperature, constant-humidity incubator maintained at 37 °C and 5% CO_2_. Six-well plates were inoculated with 1 × 10^6^ cells each and cultured under normal or starvation conditions for 24 h. Subsequently, the cells were treated with 0.5 μg/ml TM, 1.0 nM 4-PBA (both from Sigma-Aldrich, St. Louis, MO, USA) or 100 ng/ml rhIL-32β (R&D Systems Inc. - Minneapolis, USA. Catalog Number: 6769-IL).

### RNA interference

Small interfering RNA (siRNA) targeting IL-32 was achieved by introducing plasmids containing IL-32 shRNA (AGAAGCTGAAGGCCCGAATctcgagATTCGGGCCTTCAGCTTCT, targeting IL32 transcript variant1–9, which encoding five isoforms interleukin-32 namely IL-32α, IL-32β, IL-32γ, IL-32δ and IL-32ε precursor) and scrabbled shRNA (TTCTCCGAACGTGTCACGTctcgagACGTGACACGTTCGGAGAA) into A549 cells through lipofectamine 3000 transfection reagent (Invitrogen, CA, USA). The GV102 derived plasmids for shRNA and scrabbled shRNA expression were designed and provided by Shanghai Genechem Co., Ltd. (Shanghai, China). Forty-eight hours after transfection, the cells were observed under an inverted fluorescence microscope to determine the transfection rate. Subsequently, RNA extraction was performed to confirm the interference efficiency via reverse transcription-quantitative polymerase chain reaction (RT-qPCR).

### RT-qPCR

Total RNA was extracted from the A549 cells using TRIzol reagent (Takara, Japan) in accordance with the manufacturer’s instructions. Subsequently, the total RNA was reverse-transcribed to cDNA with the reverse transcription kit (Takara). Using the obtained cDNA as the template and β-actin as an internal control, RT-qPCR was performed with the real-time fluorescence-based 2 × SYBR Green qPCR mix kits (Solarbio) according to the manufacturer’s instructions. IL-32 primer design and synthesis were performed by Shanghai Genechem Co., Ltd. (Shanghai, China), and the other primers design and synthesis were performed by Sangon Biotech (Shanghai, China) and all of the sequences as follows:

IL-32 forward 5′-CGACTTCAAAGAGGGCTACC-3′ reverse 5′-GATCCTCAACATCCGGGACA-3′ (These two primers recognize the mRNA of IL32 transcript variant1–9, which encoding five isoforms interleukin-32 namely IL-32α, IL-32β, IL-32γ, IL-32δ and IL-32ε precursor).

E-Cadherin forward 5′-GGGGTCTGTCATGGAAGGTGC-3′ reverse 5′-GTAAGCGATGGCGGCATTGTA-3′.

N-Cadherin forward 5′-CATCATCATCCTGCTTATCCTGT-3′ reverse 5′-GCTCTTCTTCTCCTCCACCTTCTT-3′.

Snail forward 5′-CTTCTCCTCTACTTCAGTCTCTTCC-3′ reverse 5′-TGAGGTATTCCTTGTTGCAGTATTT-3′.

Vimentin forward 5′-AATCCAAGTTTGCTGACCTCTCTGA-3′ reverse 5′-GACTGCACCTGTCTCCGGTACTC − 3′.

Zeb-1 forward 5′-TAGATTTTGTGTGGGATTTCCTGTC-3′ reverse 5′-AGTGATTTTAATGATGGCTCGAATA-3′.

TNF-α forward 5′-AGGACACCATGAGCACTGAAAGC-3′ reverse 5′-AAGGAGAAGAGGCTGAGGAACAAG-3′.

TGF-β1 forward 5′-GAAACCCACAACGAAATCTATGAC-3′ reverse 5′-ACGTGCTGCTCCACTTTTAACT-3′.

IL-1β forward 5′-GAAATGATGGCTTATTACAGTGGCA-3′ reverse 5′-GTAGTGGTGGTCGGAGATTCGTAG-3′.

IL-6 forward 5′-CCTCCAGAACAGATTTGAGAGTAGT-3′ reverse 5′-GGGTCAGGGGTGGTTATTGC-3′.

β-actin forward 5′-CCCATCTATGAGGGTTACGC-3′ reverse 5′- TTTAATGTCACGCACGATTTC-3′.

### MTT assay

A549 cells in logarithmic growth phase were seeded into 96-well plates at a density of 5, 000 cells/well. Cells were divided into blank control group, solvent group (DMSO) and tunicamycin (0.5 μmol/L) group and cultured in a humidified chamber at 37 °C for 0, 24, 48 h. Viable cells were evaluated with MTT assay kit (Sigma, USA) according to the manufacturer’s instructions. Briefly, 20 μL MTT (5 mg/mL) solution was added to each well and the plates were incubated at 37 °C for 4 h. The absorption value of every well was read at 490 nm using a microplate reader (ELX800, Bio-Tek, USA).

### ELISA assay

The supernatants of A549 cells culture were collected and the concentration of TNF-α, TGF-β1, IL-1β, IL-6 in the supernatants was determined by using human TNF-α, TGF-β1, IL-1β, IL-6 ELISA Kit (Neobioscience, Shenzhen, Guangzhou, China) following the manufacturer’s instructions.

### Western blotting

Total protein was extracted from the A549 cells and quantified using the BCA protein assay kit (Solarbio, Beijing, China). A sample containing 20 μg of protein was separated on a 12% sodium dodecyl sulfate-polyacrylamide gel electrophoresis gel (Solarbio), transferred to a polyvinylidene fluoride membrane, and incubated with rabbit anti-mouse N-cadherin, GRP78, and α-SMA primary antibodies (Proteintech, Wuhan, China), or β-actin antibody (Bioss, Beijing, China) as a loading control, at room temperature for 2 h. Subsequently, the membrane was washed, incubated with the horseradish peroxidase-conjugated goat anti-rabbit secondary antibody (Proteintech, Wuhan, China) for 1 h at room temperature, and exposed using the ECL kit. The experiment was repeated three times.

### Statistical analysis

SPSS 20.0 software was used for statistical analysis of the experimental data. Quantitative data are expressed as the mean ± standard deviation, *n* = 3, and comparisons between two groups were performed using the t-test. A significance level of α = 0.05 was adopted, with *P < 0.05* indicating a statistically significant difference.

## Results

### Tunicamycin treatment enhanced the expression of IL-32 in A549 cells

**Fig. 1:**
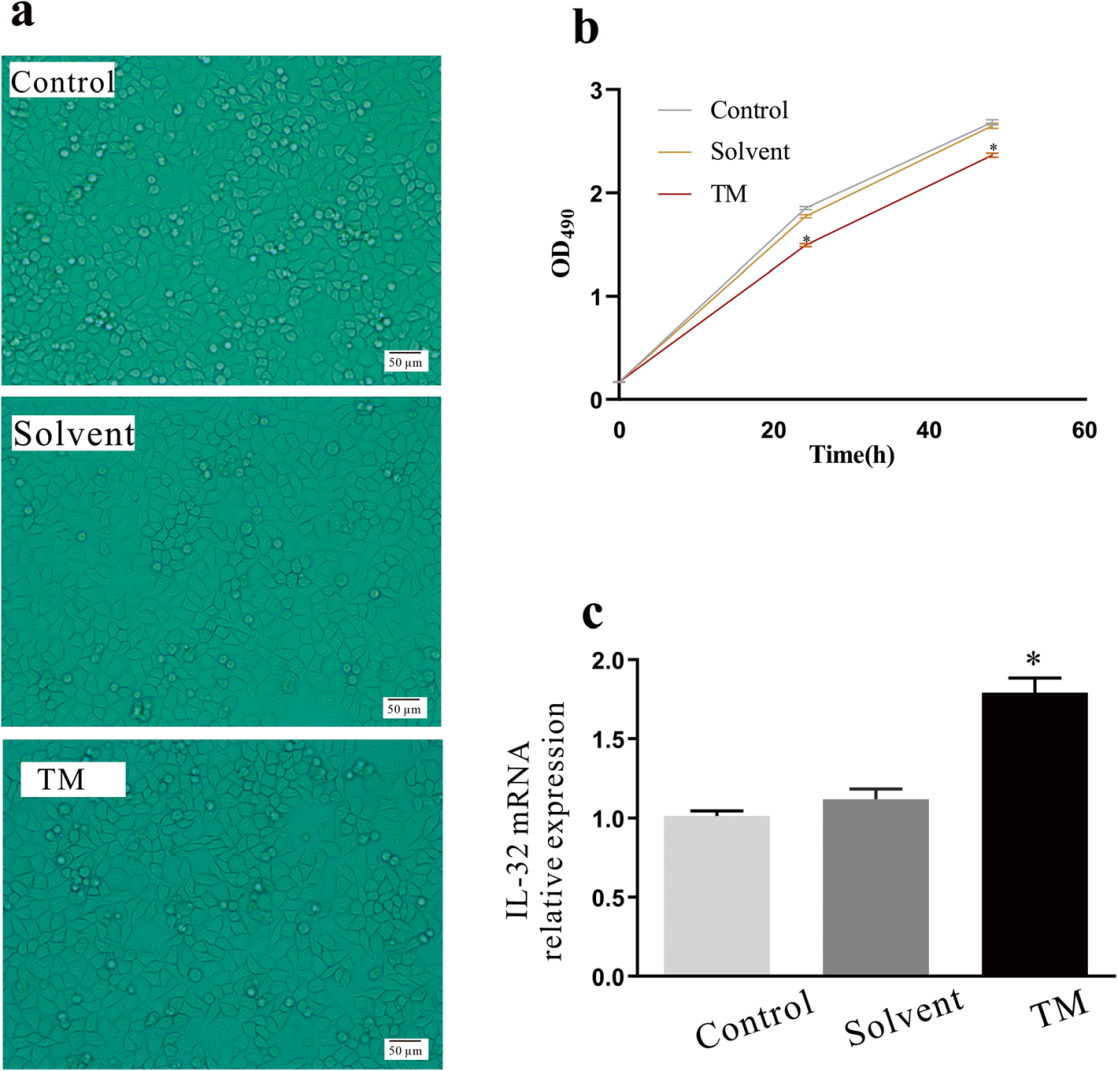
Morphology and IL-32 expression changes of TM treatment. **a** Morphologies of A549 cells of the control, solvent (DMSO)-treated, and TM-treated groups. **b** MTT assay of cell proliferation of control, solvent-treated, and TM-treated cells. **c** mRNA expression levels of IL-32, measured by RT-qPCR. * *P < 0.05* compared with the control or solvent group

Our previous study showing that TM can induce EMT in A549 cells, the cells were treated with 0.5 μg/ml TM for 24 h [11]. Observation of the cells under an inverted microscope showed that the TM treatment reduced the cell number compared with that of the control and solvent group (Fig. 1a). Although the TM-treated cells showed no significant morphological changes, enlargement of intercellular spaces was evident, resulting in a looser arrangement of cells (Fig. 1a). Meanwhile, an MTT assay was applied to evaluate the effect of TM treatment on cell proliferation. The result showed that compared with the control group and the solvent group, TM treatment reduced the cell proliferation ability of the cells, resulting in a significant reduction in the number of cells observed under the microscope (Fig. 1b). This is consistent with the previous report that ETM led to cell cycle arrest in G1 phase regulated by increased levels of p21Waf1/Cip1 [17]. Moreover, RT-qPCR showed that the mRNA expression level of IL-32 was significantly increased (*P < 0.05*) after 24 h of TM stimulation compared with the control or solvent group, while the levels between solvent (DMSO) treated group and control group showed no statistically significance (Fig. 1c).

### rhIL-32 induced EMT in A549 cells

**Fig. 2:**
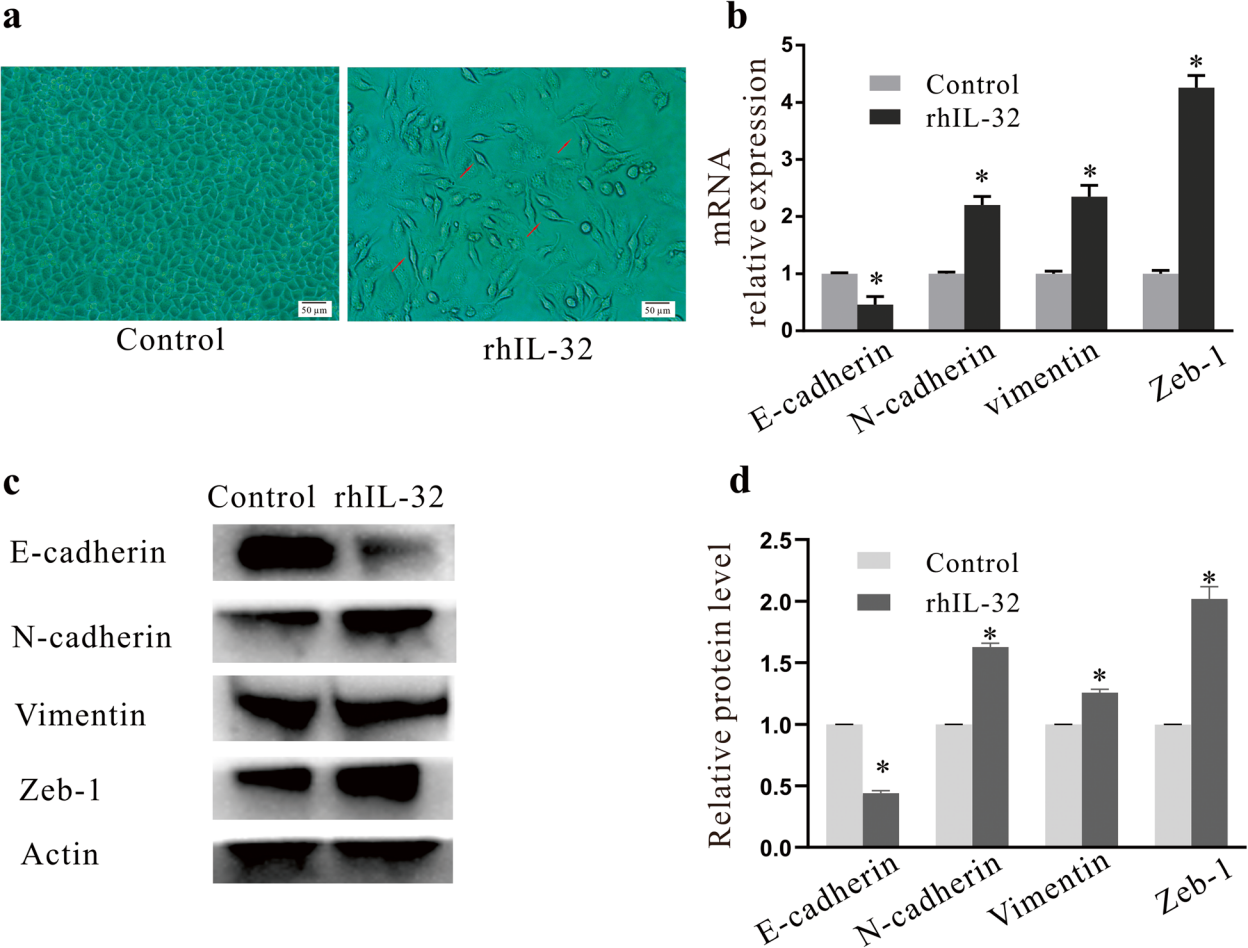
Morphology and EMT-related gene expressions in rhIL-32 treated cells. **a** Morphologies of A549 cells of the control and rhIL-32 treated groups observed under an inverted microscope. **b** mRNA expression levels of EMT-related molecular markers, measured by RT-qPCR. **c**, **d** Protein expression levels of EMT-related molecular markers measured by western blotting. *P < 0.05 compared with the control group

After treatment with 100 ng/ml rhIL-32 for 24 h, a portion of the A549 cells showed a morphological change from a pebble-like shape to an irregular elongated shape (indicated by the red arrow in Fig. 2a). To ascertain if this phenomenon was caused by EMT, RT-qPCR was adopted to determine the influence of rhIL-32 treatment on the expression of EMT-related molecules. Indeed, compared with the blank control expression of the epithelial cell marker E-cadherin was significantly downregulated (*P < 0.05*, Fig. 2b, c, d), while the expression of the mesenchymal cell markers N-cadherin, Vimentin, and Zeb-1 were significantly upregulated (*P < 0.05*, Fig. 2b, c, d) after rhIL-32 treatment.

### IL-32 siRNA inhibited TM-induced EMT

**Fig. 3:**
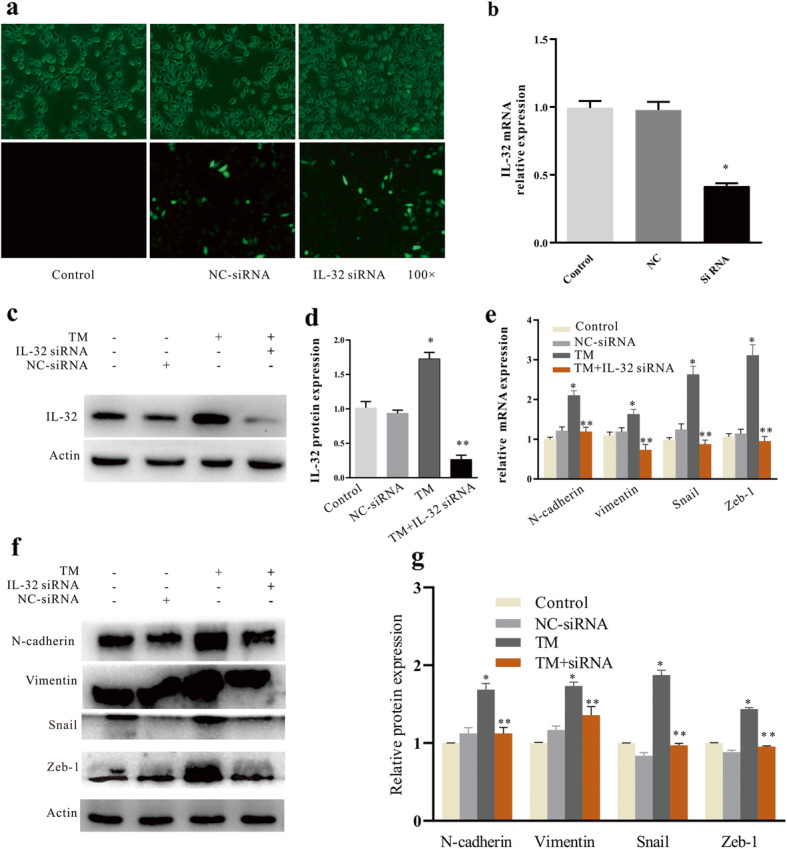
Inhibition of EMT in A549 cells by IL-32 siRNA. **a** Transfection efficiency of A549 cells: A549 were cultured in vitro and inoculated onto six-well plates. When the cells reach confluence, Lipofectamine 3000 transfection reagent was used to transfect A549 cells with fluorescent plasmids containing IL-32 siRNA. Forty-eight hours after transfection, the IL-32 shRNA transfection was evaluated by observing the intensity of green fluorescent protein with an inverted fluorescence microscope. The IL-32 gene silencing efficiency was measured using RT-qPCR **b** and western blotting assay **c**, **d**. **e** The mRNA expression levels of EMT-related molecular markers after transfection with IL-32 siRNA. *P < 0.05 compared with the control group, **P < 0.05 compared with the TM group. **f**, **g** The protein expression levels of EMT-related molecular markers after transfection with IL-32 siRNA. *P < 0.05 compared with the control group, **P < 0.05 compared with the TM group

Forty-eight hours after the transfection of A549 cells with IL-32 siRNA, the transfection rate observed under an inverted fluorescence microscope was estimated at approximately 50–60% (Fig. 3a). RT-qPCR (Fig. 3b) and WB (Fig. 3c, d) were used to detect the interference rate of IL-32. Compared with TM group, the expression of TM + IL-32 siRNA group decreased significantly (*P < 0.05*). The A549 cells were divided into the control, Lipo™3000/NC (NC), TM, and TM + IL-32 siRNA groups (TM was added 24 h after siRNA transfection). RT-qPCR showed that the mRNA expression levels of the mesenchymal cell markers N-cadherin, Vimentin, Snail, and Zeb-1 were significantly increased in the TM group compared with those of the control group (*P < 0.05*, Fig. 3e, f, g), whereas these expression levels were significantly decreased in the TM + IL-32 siRNA group compared with those of the TM group (*P < 0.05*, Fig. 3e, f, g), further demonstrating that IL-32 interference inhibits TM-induced EMT.

### rhIL-32 induced ER stress in A549 cells

**Fig. 4:**
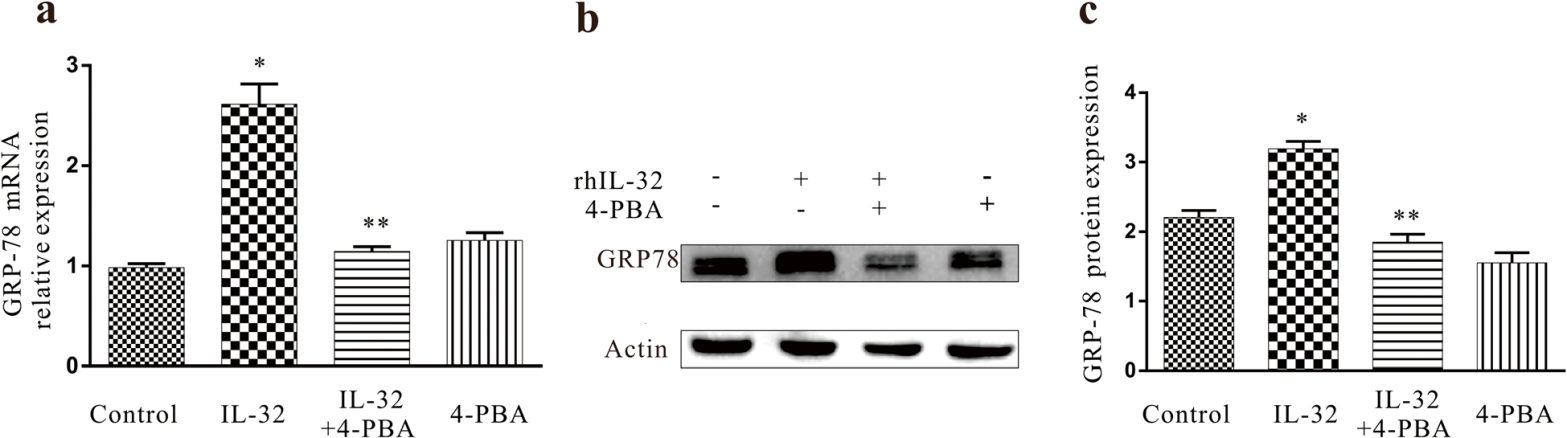
GRP78 expression characteristics induced by IL-32 or 4-PBA. **a** GRP78 mRNA expression levels. *P < 0.05 compared with the control group, **P < 0.05 compared with the rhIL-32 group. **b** and **c** GRP78 protein expression levels, measured by western blotting. *P < 0.05 compared with the control group, **P < 0.05 compared with the rhIL-32 group

Twenty-four hours after treatment of A549 cells with 100 ng/ml rhIL-32, both the mRNA and protein expression levels of GRP78 increased significantly (*P < 0.05*, Fig. 4a, b, c), indicating that rhIL-32 can induce ER stress in A549 cells.

Moreover, treatment of rhIL-32 with 1.0 mM 4-PBA significantly decreased the mRNA and protein expression levels of GRP78 compared with those of cells treated with rhIL-32 alone (*P < 0.05*, Fig. 4a, b, and c).

### ER stress mediated EMT in A549 cells

The aforementioned results suggest that rhIL-32 induces both EMT and ER stress in A549 cells. To further ascertain the relationship between ER stress and EMT, the expression of EMT-related molecules was measured in A549 cells treated with the ER stress inhibitor 4-PBA.

**Fig. 5:**
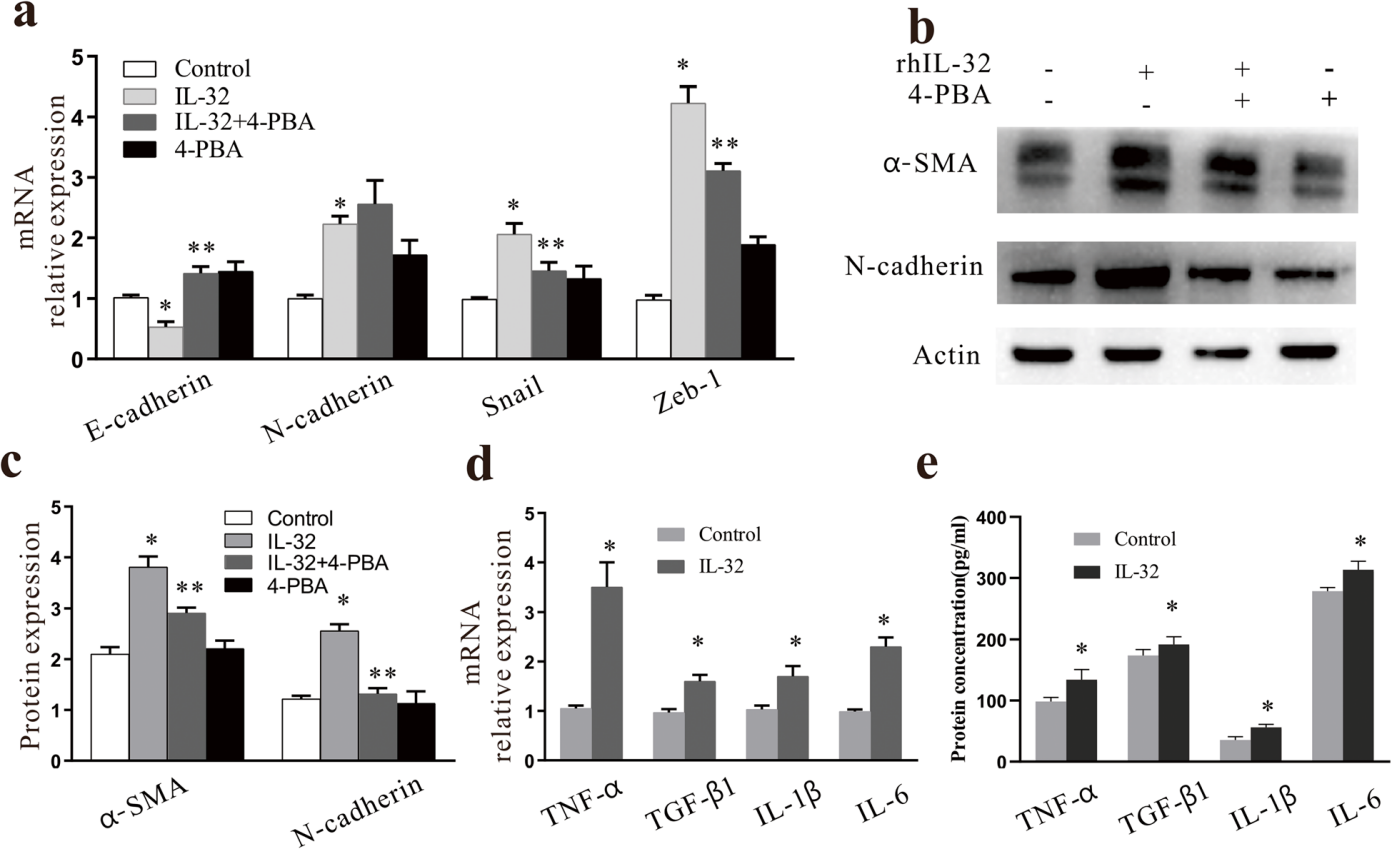
The effect of IL-32 or 4-PBA on the expression of EMT related genes and effect of IL-32 on the expression of inflammatory cytokine. **a** mRNA expression levels of EMT-related marker genes. * *P < 0.05* compared with the control group, ** *P < 0.05* compared with the rhIL-32 group. **b** and **c** Protein expression levels of EMT-related proteins measured by western blotting. *P < 0.05 compared with the control group, **P < 0.05 compared with the rhIL-32 group. **d** mRNA expression of inflammatory cytokines in control and rhIL-32 treated A549 cells. *P < 0.05 compared with the control group. **e** Protein expression of inflammatory cytokines in control and rhIL-32 treated A549 cells measured by ELISA assay. *P < 0.05 compared with the control group

Compared with cells treated with rhIL-32 alone, 4-PBA treatment reduced the mRNA expression levels of the mesenchymal cell markers N-cadherin (*P < 0.05*), snail (*P < 0.05*), and Zeb-1 (*P < 0.05*) (Fig. 5a), and increased the mRNA expression level of the epithelial cell marker E-cadherin (*P < 0.05*, Fig. 5a). Similarly, the protein expression of N-cadherin and α-SMA were upregulated in the rhIL-32 group but significantly downregulated in the rhIL-32 + 4-PBA group (*P < 0.05*, Fig. 5b, c). Thus, EMT was inhibited following the inhibition of ER stress, indicating that rhIL-32 triggers ER stress and mediates the development of EMT in A549 cells.

### IL-32 induces inflammatory cytokine production in A549 cells

After rhIL-32 treatment, the mRNA expression levels of the inflammatory cytokines tumor necrosis factor (TNF)-α, transforming growth factor (TGF)-β1, IL-1β, and IL-6(Fig. 5d) in the rhIL-32 group were significantly higher than those of the control group *(P < 0.05*). An ELISA assay was further employed to measure the protein levels of these inflammatory cytokines, and the result was consistent with the RT-PCR results. These results indicate that IL-32 could induce inflammatory cytokine production in A549 cells.

## Discussion

IPF is a heterogeneous process involving the participation of multiple factors. In particular, EMT in pulmonary cells is one of the key pathogenic mechanisms of the disease, as myofibroblasts are mainly formed from the transition of pulmonary alveolar epithelial cells to mesenchymal cells. However, at present, the mechanisms by which EMT occurs in pulmonary epithelial cells remain unclear.

Interleukin (IL)-32, a well-known cytokine, has nine alternative spliced isoforms i.e. IL-32α, IL-32β, IL-32γ, IL-32δ, IL-32η, IL-32θ, IL-32ε, IL-32ζ, IL- 32ι [18, 19], and IL-32α, IL-32β, IL-32γ and IL-32δ were the four major isoforms isolated from IL-2-stimulated human NK cells [20]. IL-32 is associated with inflammation, virus infections and cancer by participating in multifaceted regulation process such as cancer cell growth inhibition, cell apoptosis regulation, accentuation of inflammation, and angiogenesis [13, 15]. Several recent studies have pointed to a critical role of IL-32 in the onset and development of EMT in cells. Using Vimentin as an EMT marker, Su et al. found that IL-32β can induce Slug and Vimentin expression in breast cancer cells, thereby proving that IL-32 causes the onset of EMT in breast cancer cells [14]. Similarly, Lin et al. found that IL-32θ can inhibit EMT in colon cancer stem cells through the STAT3 signaling pathway [15]. Other researchers have reported that IL-32α can inhibit the JAK2/STAT3 signaling pathway and reverse the IL-6-induced EMT process in pancreatic cancer cells [13]. The present study confirms and expands this role of IL-32, in demonstrating that it could induce EMT in pulmonary alveolar epithelial cells.

The process by which ER stress and unfolded protein response-mediated EMT occurs [10, 21–23] plays a critical role in the pathogenesis of fibrosis in many organs [24–27]. Some studies have found that ER stress induced by TM, thapsigargin, and mutations in *SPC* and *SPA* can mediate the development of EMT in pulmonary alveolar epithelial cells [9, 10]. In addition, bleomycin could induce the onset of ER stress in a mouse model of pulmonary fibrosis, which in turn mediated the occurrence of EMT [28]. A similar conclusion was obtained in the present study, as IL-32 induced EMT through the induction of ER stress in pulmonary alveolar epithelial cells.

A multitude of factors have been suggested to trigger ER stress and consequently induce the development of EMT in pulmonary alveolar epithelial cells, including genetic and environmental factors such as smoking, chronic aspiration, viral infection, polymorphism in the promoter region of mucoprotein 5B, telomerase gene mutations, and mutations in *SPC* and *SPA* [24, 29, 30]. However, the exact mechanisms remain unclear. Although IL-32 appears to play a role in this process, we have not made clear which subtype of IL-32 plays a key role in it, in-depth investigation of these mechanisms and the role of each subtype of IL-32 will be the next step in our research.

The ER stress inhibitor 4-PBA was reported to induce EMT by TM-induced ER stress in renal tubular epithelial cells [31], and could also inhibit the expression of the EMT downstream markers GRP78 and LC3B-II in breast cancer cells [32]. Moreover, 4-PBA can alleviate atherosclerosis and stabilize existing plaques in mice through inhibition of ER stress [33]. Although similar effects of 4-PBA were observed in the present study by inhibiting TM-induced EMT, N-cadherin expression was not significantly reduced compared with the rhIL-32 treatment group. This could be attributed to the relatively short time of 4-PBA treatment in the experiment, which may have been insufficient for 4-PBA to exert its inhibiting effects on N-cadherin expression.

In the development of pulmonary fibrosis, the expression of profibrotic and antifibrotic cytokines are upregulated and downregulated, respectively, thereby causing an imbalance. Previous studies have indicated that multiple cytokines, including TGF-β1, IL-1β, TNF-α, IL-17, IL-27, IL-13, and IL-32, are overexpressed in pulmonary fibrosis [34–37]. In addition, ER stress can promote the protein expression of LTBP1 and LTBP4, which are closely related to the secretion of TGF-β1 and TGF-β4 in pulmonary epithelial cells, thereby playing a key role in the development of pulmonary fibrosis [38]. The present study showed that rhIL-32 can induce ER stress and consequently upregulate TGF-β1, TNF-α, IL-6, and IL-1β expression. Because the high sequences similarity between interleukin-32 transcript variant 1–9 which encoding IL-32α, IL-32β, IL-32γ, IL-32δ and IL-32ε, and there are limited commercially available isoforms of recombinant protein of IL-32. We only examined the effects of IL-32β on EMT, ER stress and cytokine production. Whether the IL-32α, IL-32γ, IL-32δ and IL-32ε exhibit similar functions remains to be further studied. Meanwhile, further research will also be conducted to determine which upstream pathways are involved in the regulation cytokine overexpression.

## Conclusion

This study found that IL-32 can induce EMT in pulmonary alveolar epithelial cells by triggering ER stress offers a new approach for studies on IPF. In future research efforts, we will conduct an in-depth investigation of the relevant mechanisms in an attempt to establish IL-32 as a novel target for the treatment of pulmonary fibrosis.

## Supplementary information

**Additional file 1.** Figure S1. Flow cytometry analysis of cell apoptosis of the blank group (a), solvent group (b), and TM treated group (c)

## Data Availability

All data generated or analyzed during this study are included in this published article.

## Abbreviations

EMT: Epithelial-mesenchymal transition
IPF: Idiopathic pulmonary fibrosis
ER Stress: endoplasmic reticulum stress
IL-32: Interleukin-32
rhIL-32β: Recombinant human interleukin-32 beta protein
siRNA: Small interfering RNA
4-PBA: 4-phenylbutyric acid
RT-qPCR: Reverse transcription-quantitative polymerase chain reaction
GRP78: Glucose-regulated protein 78
TM: Tunicamycin
WB: Western blotting
DMSO: Dimethyl sulfoxide
α-SMA: Alpha smooth muscle Actin
Zeb-1: Zinc finger E-box binding homeobox 1
